# A novel technique for avoidance of sternotomy, diaphragmic incision and cardiopulmonary bypass during cavoatrial tumor thrombectomy for renal cell carcinoma with intraatrial tumor thrombus: a case series at a single center

**DOI:** 10.1186/s12893-023-02156-7

**Published:** 2023-08-24

**Authors:** Wei He, Zixiang Cong, Yaqing Liu, Zhongshun Yao, Fajuan Cheng, Yiming Zhang, Zhihong Niu

**Affiliations:** 1 Department of Urology, Shandong Provincial Hospital Affiliated to Shandong First Medical University, 324, Jingwu Road, Huaiying District, Jinan, 250021 Shandong China; 2 Department of Urology, Shandong Provincial Hospital, Shandong University, Jinan, Shandong province China; 3 Department of Nephrology, Shandong Provincial Hospital Affiliated to Shandong First Medical University, Jinan, Shandong China

**Keywords:** Renal cell carcinoma, Surgery, Atrium, Tumor Thrombus, Cardiopulmonary bypass

## Abstract

**Background:**

Radical nephrectomy with thrombectomy in patients with renal cell carcinoma (RCC) and level IV thrombus extending to the right atrium (RA) offers improved survival. However, this procedure is associated with significant perioperative morbidity and mortality. In this report, we describe a novel milking technique for patients with RA tumor thrombus using abdominal access, which does not require diaphragmic incision, sternotomy, right atriotomy, or cardiopulmonary bypass (CPB).

**Methods:**

Between January 2019 and January 2022, four patients underwent resection of renal cell carcinoma extending into RA by a milking technique developed to avoid diaphragmic incision, sternotomy, or CPB. Patient characteristics, perioperative data, pathological features, and survival were evaluated.

**Results:**

Complete resection was successful through pure transabdominal access without diaphragmic incision, sternotomy, or CPB in all patients.

**Conclusion:**

We conclude that radical nephrectomy and thrombectomy in optimized cases with renal cell carcinoma extending into RA can be safely and effectively performed without diaphragmic incision, sternotomy, or CPB, avoiding serious perioperative complications while providing acceptable oncological outcomes.

## Background

Tumor thrombus extending to the inferior vena cava (IVC) has been reported in 4–10% of renal cell carcinoma (RCC) cases [1]. Complete surgical removal of renal mass and thrombus is the standard treatment for selected patients as it offers significant survival benefits [2]. However, the thrombectomy for patients with an extension of tumor tissue into the right atrium (RA) represents the most critical surgical challenge. In this scenario, the open approach is generally favored. The surgical management consists of sternotomy, right atriotomy, and cardiopulmonary bypass (CPB) with deep hypothermic circulatory arrest (DHCA) [3, 4]. Although this procedure provides a bloodless field and clear visualization for thrombus removal, it significantly prolongs the operative time and is associated with more significant mortality and morbidity [5]. Recent studies have suggested that supradiaphragmatic tumor thrombus (Mayo IV) can be removed without sternotomy and any form of CPB with decreased morbidity and low mortality. However, these surgeries were completed by dissection of the central tendon and pericardium to expose the IVC and RA [6–8]. Although the open approach is generally favored, minimally invasive approaches have been successfully applied using robot-assisted laparoscopic techniques by surgeons with expertise. The intrapericardial IVC control is achieved through transabdominal incision of the central tendon of the diaphragm and the pericardium in both open and minimally invasive surgery. Here, we describe a novel technique for patients with RA tumor thrombus using abdominal access, for which proximal control can be gained without diaphragmic incision, sternotomy, right atriotomy, or CPB.

## Methods

### Patients

After approval by the Institution Review Board (SWYX: No.2022-80), we retrospectively reviewed the surgical records of patients with renal cell carcinoma and tumor thrombus extending to the right atrium in our center from January 2019 to January 2022. Patients treated by transabdominal radical nephrectomy and thrombectomy without sternotomy, diaphragmic incision, or cardiopulmonary bypass were included. Four patients were identified. Patients’ characteristics are summarized in Table 1.

**Table 1:** Characteristic and clinical data of 4 patients

	Patient			
Variable	1	2	3	4
Age, years	52	63	75	57
Body mass index, kg/m2	26.9	27.3	25.3	21.1
Sex	Male	Female	Female	Male
ECOG performance status	1	0	1	1
TNM stage	pT3cN0M0	pT3cN0M0	pT3cN0M0	pT4N1M0
Tumor, cm	9.8	8.2	5	9
Tumor thrombus in RA, cm	3.2	3.7	4.3	0.5
Operative time, minutes	180	160	220	250
Contorl of porta hepatis, minutes	28	14	20	30
Estimated blood loss, ml	200	300	350	800
Blood transfusion, units	0	0	0	6
ICU stay	1	2	2	1
Day to surgical drain removed	3	4	4	4
Postoperative complications			Lymphatic leakage	Pleural effusion, pulmonary infection
Postoperative serum Creatinine, µmol/L	79	48	105	74
Postoperative serum ALT, u/L	77	109	68	26
Postoperative serum AST, u/L	99	163	90	35
Postoperative hospitalization	7	7	8	8
Pathology	ccRCC	ccRCC	ccRCC	ccRCC
WHO/ISUP grade	2	2	3	3
Survival state	Alive	Alive	Alive	Alive
Survival time, months	36	13	12	5

ALT, alanine aminotransferase; AST, aspartate aminotransferase; ccRCC, clear cell renal cell carcinoma; ECOG, Eastern Cooperative Oncology Group; ICU, intensive care unit; WHO, World Health Organization; ISUP, International Society of Urological Pathology; RA, right atrium; TNM, tumor nodes metastasis staging system.

**Fig. 1:**
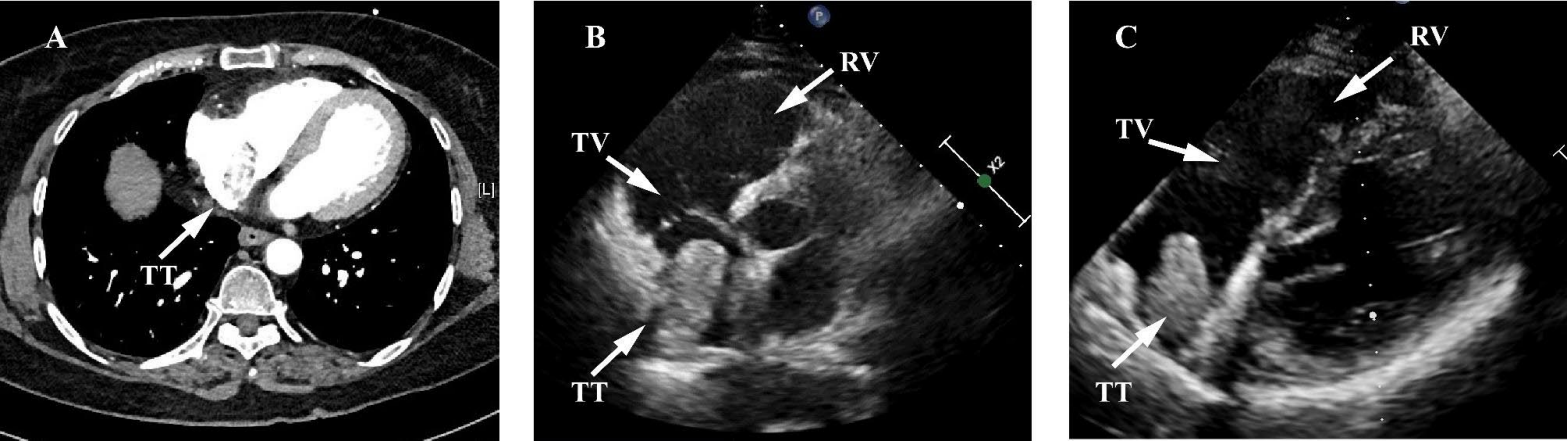
The preoperative imaging findings of renal cell carcinoma patients with right atrial tumor thrombus. (**A**) Contrast-enhanced computed tomography demonstrating the tumor thrombus (white arrow) in the right atrium. (**B**, **C**) The ultrasonography showing the tumor thrombus floating in the right atrium. RV, right ventricle; TT, tumor thrombus; TV, tricuspid valve

All the patients had preoperative computed tomography or magnetic resonance imaging scans. The imaging modalities demonstrated renal cell carcinoma with tumor thrombus extending to the right atrium (Fig. 1). The thrombi mobility and involvement with the vena cava were assessed by abdominal Doppler ultrasonography. Three patients had right-sided renal cell carcinoma. One patient with a left-sided renal mass had synchronous metastasis and received pazopanib treatment for six months. Routine laboratory investigations showed no abnormality. The functions of vital organs were evaluated prior to operations. All the patients’ performance statuses were evaluated according to the Eastern Cooperative Oncology Group criteria. No preoperative renal artery embolization was performed in any patients. Intraoperative real-time transesophageal echocardiography (TEE) was utilized to monitor thrombectomy. Postoperative follow-up evaluations included physical examinations, laboratory tests, and imaging modalities.

### Surgical technique

After the induction of general anesthesia, the patients were placed in the supine position with both groins exposed. A continuous intraoperative TEE probe was inserted to delineate the cranial extent and adherence of the tumor thrombus.

IVC exposure and renal artery ligation: For patients with right renal mass (patients 1 through 3), the laparotomy incision was extended two fingerbreadths below the right costal margin from the xiphoid process to the lateral midaxillary line. When the right colon was mobilized medially, the duodenum was dissected posteriorly and was mobilized medially over to the aorta (Kocher maneuver). The IVC was gently exposed from the third hepatic portis to the level below the renal vein. As the right kidney was mobilized laterally and posteriorly, the right renal artery was encountered, ligated, and divided.

For patient 4 with left RCC, the surgical technique of transperitoneal laparoscopic radical nephrectomy was performed using routine procedures [9]. The left renal vein with thrombus was secured and divided *en bloc* with the firing of the Endo-GIA stapler.

**Fig. 2:**
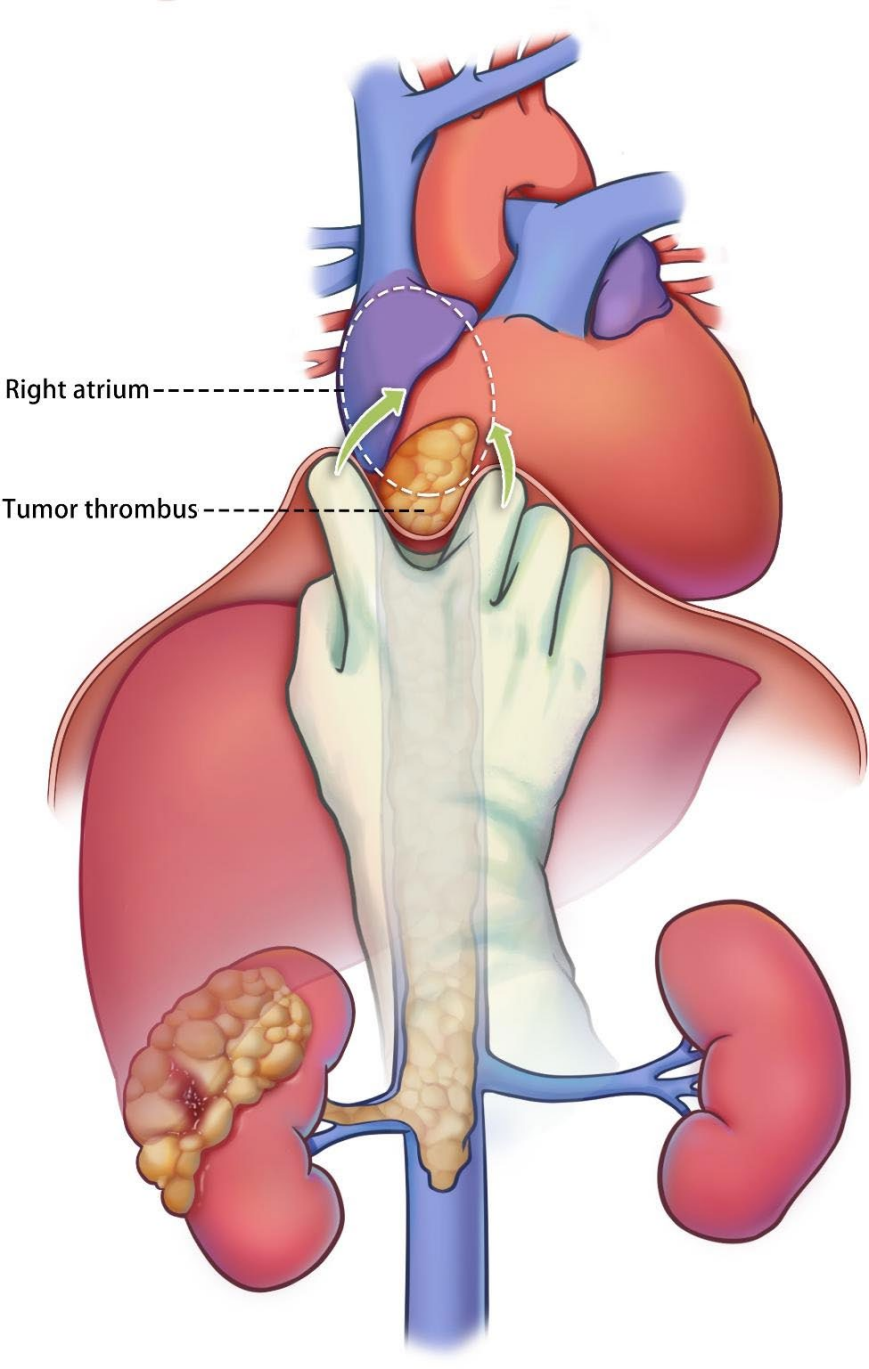
Abdominal control of renal cell carcinoma with right atrial tumor thrombus without diaphragmic Incision and cardiopulmonary bypass. The diaphragm can be raised to the cranial along with the IVC, and then the RA can be squeezed from both sides through the diaphragm. The tumor thrombus in RA was then “milked” down to the IVC

Liver mobilization and IVC clamping: After the ligamentum teres was ligated and divided, the falciform ligament was incised. The coronary ligament and diaphragm around the vena cava hiatus were dissected circumferentially with satisfactory mobility. The distal IVC and the contralateral renal vein were blocked. The porta hepatis was controlled using Pringle’s maneuver. After dissection around the vena cava hiatus, the diaphragm can be raised to the cranial along with the IVC, and then the RA can be gently squeezed from both sides through the diaphragm. The tumor thrombus in RA was then “milked” down to the IVC (Fig. 2). The Satinsky clamp was placed across the intrapericardial IVC under TEE monitoring without sternotomy, diaphragmic incision, or cardiopulmonary bypass.

Thrombectomy: After achieving reliable vascular control, longitudinal cavotomy was performed around the renal vein ostium. Digital manipulation or withdrawal maneuvers were used to remove the tumor thrombus. After complete resection, the cavotomy was closed with 4 − 0 PROLENE™ Polypropylene running sutures. Finally, a drain was placed within the peritoneal space, and the laparotomy incision was closed layers. One patient (patient 4) required the excision of a segment of the infrahepatic IVC wall, which was repaired using a bovine pericardium patch.

## Results

These four patients’ characteristics and clinical data are presented in Table 1. The median size of the renal mass was 8.2 cm. The median length of the tumor thrombus in RA was 3.2 cm. All the operations were performed successfully. No perioperative death occurred. The median operative time was 180 min. The median duration of porta hepatis control by a Rummel tourniquet was 20 min. The median estimated blood loss was 300 ml. Only patient 4 required the perioperative transfusion of 6 units of blood and 760 ml plasma due to infrahepatic IVC involvement and reconstruction. The median length of intensive care unit stay was one day. The median drainage duration was four days. The median values of postoperative serum creatinine was 74 µmol/L. The median value of postoperative alanine aminotransferase and aspartate aminotransferases were 68 and 90 u/L, respectively. There was one patient who developed lymphatic leakage. One patient suffered postoperative pulmonary infection and pleural effusion. Based on the Clavien-Dindo classification of surgical complications, these patients’ complications were grade 2. The main reason was the trauma caused fluid collection in the surgical site during the operation. There were no immediate or 30-day postoperative deaths. The median length of postoperative hospitalization was seven days. The tumor pathology analysis demonstrated clear cell renal cell carcinoma in all patients. According to the WHO/ International Society of Urological Pathology grading system, two cases were classified as 2 and two as 3. During the follow-up period, all the patients were still alive at the time of this report. It was also noted that no recurrence was found in any patient.

## Discussion

Here, we report 4 RCC patients with right atrial tumor thrombus that could be “milked” into the IVC without applying CPB and DHCA. This approach is safe and feasible. Atrial thrombosis is a relatively rare event in about 1% of patients affected by renal cell carcinoma. Radical nephrectomy and thrombectomy in patients with renal cell carcinoma and RA tumor thrombus extension offer them a chance of long-term survival [10–12]. The standard approach to such entities has been that of CPB with DHCA. Although these techniques provide satisfactory visualization for tumor thrombus dissection and rapid intervention for embolism in case of thrombus splintering, the limitation of CPB and DHCA is the increased complication and morbidity, such as transient or permanent neurologic deficits, including delirium and stroke [13, 14]. Besides, the disorder in coagulation and hepatic dysfunction also lead to postoperative bleeding [15–17]. Moreover, ischemic injury to the vital organs, as well as infection secondary to ischemic reperfusion injury, compromise recovery. Overall mortality has been reported to be up to 10% [13, 14]. If the thrombectomy in cases of RCC with RA tumor thrombus could be performed without CPB or DHCA, the patients may undergo tumor excision with minimal morbidity and mortality.

In the past few decades, urologists and cardiac surgeons have tried to carry out radical nephrectomy and supradiaphragmatic tumor thrombus to avoid CPB or DHCA. Stewart and colleagues reported their experience with eight patients who underwent cavoatrial thrombectomy in whom DHCA was not used [16]. CPB was established routinely. They employed cardiotomy suction to aspirate coronary and hepatic venous to provide a virtually bloodless field. However, this technique may facilitate malignant cell spreading. Ruel et al. [18]. and Shinghal et al. [19]. reported novel techniques involving CPB and clamping the descending aorta above the diaphragm with avoidance of DHCA. These techniques were associated with several advantages. Occlusion of the descending aorta near the diaphragm diminished low body and viscera venous return, providing bloodless exposure during cavoatrial thrombus excision and avoiding the need for DHCA. Cerebral and spinal cord perfusion was maintained; thus, the risk of transient or permanent neurologic damage was reduced.

Recently, many investigators have reported a transabdominal approach to level IV and RA tumor thrombi that decreases morbidity and mortality associated with CPB and DHCA. Cinacio and colleagues [6, 20] described a liver transplantation technique that avoided complications that were associated with CPB. After having a full dissection of the liver to gain access to the intrahepatic, suprahepatic IVC, the liver was rolled to the left. The central diaphragm tendon was dissected circumferentially to identify the supradiaphragmatic, intrapericardial IVC. The RA was then softly pulled beneath the diaphragm into the abdomen. In case of more exposure to RA was needed, the central tendon was incised to expose the pericardium, and a pericardiotomy was performed. The tumor thrombus was then milked down below the diaphragm under the TEE monitoring, allowing to clamp the IVC subdiaphragmatically. However, this milking maneuver on tumor thrombus remains disputed, as it may contribute to pulmonary embolization, metastasis, and caval tears.

In this study, we describe a transabdominal approach to tumor thrombus extending to the RA, avoiding the mortality, morbidity, and disadvantages associated with CPB and DHCA. This approach is simple, feasible, and minimally invasive compared with the alternatives mentioned above. After the ligamentum teres and falciform ligament are incised. We only dissected the coronary ligament and diaphragm around the vena cava hiatus circumferentially with satisfactory mobility. The diaphragm can be raised to the cranial along with the IVC, and then the RA can be squeezed from both sides through the diaphragm. The tumor thrombus in RA was then “milked” down to the IVC. No diaphragmic incision, sternotomy, or right atriotomy is necessary. Moreover, transfusion requirements are minor compared to the alternatives mentioned earlier. Consequently, the operation is simplified, duration is significantly reduced, and complication is decreased.

We report a feasible and safe modality using a transabdominal approach, avoiding diaphragmic incision, sternotomy, right atriotomy, or CPB, representing an alternative technique in the evolutionary management of RCC with RA tumor thrombus. Avoidance of diaphragmic incision, sternotomy, and right atriotomy shortens and simplifies the procedure as acknowledged. This technique is not always feasible, mainly when the tumor thrombus infiltrates the IVC wall. However, with well-planned surgical tactics, we believe that this approach is viable and safe in well-selected patients to avoid the potential sequelae from CPB and DHCA. The methodological limitations of the present study include the lack of a control group, its small sample size, and its retrospective nature with limited follow-up. Ideally, a prospective randomized controlled trial with large cohorts is needed to investigate the novel milking technique for patients with renal cell carcinoma and a level IV thrombus extending to the right atrium.

## Conclusion

Radical nephrectomy and cavoatrial thrombus excision of the perioperative optimized patients with RCC and RA tumor thrombus can be performed with transabdominal control of tumor thrombus. Diaphragmic incision, sternotomy, right atriotomy, and CPB are, therefore, not imperative, and these avoidances may simplify the operation and provide acceptable cancer control and mortality results. The preliminary data suggests this approach has an encouraging safety profile and provides a surgical option for patients with RCC and RA tumor thrombus. This technique is applicable for well-selected patients. More evidence concerning the favorable role of this procedure will be elucidated in further studies.

## Data Availability

The datasets used and/or analyzed during the current study available from the corresponding author on reasonable request.

## Abbreviations

CPB: cardiopulmonary bypass
DHCA: deep hypothermic circulatory arrest
IVC: inferior vena cava
RA: right atrium
RCC: renal cell carcinoma
TEE: transesophageal echocardiography
TM: trademark
